# Thermal and optical properties of PMMA films reinforced with Nb_2_O_5_ nanoparticles

**DOI:** 10.1038/s41598-021-01282-7

**Published:** 2021-11-18

**Authors:** B. Hajduk, H. Bednarski, P. Jarka, H. Janeczek, M. Godzierz, T. Tański

**Affiliations:** 1 Centre of Polymer and Carbon Materials, Polish Academy of Sciences, 34 Marie Curie-Skłodowska str., 41-819 Zabrze, Poland; 2 Department of Engineering Materials and Biomaterials, Silesian University of Technology, 18a Konarskiego str., 41-100 Gliwice, Poland

**Keywords:** Chemical engineering, Materials chemistry, Organic chemistry, Physical chemistry, Polymer chemistry

## Abstract

The article presents the thermal and physical properties of PMMA composite films with the addition of Nb_2_O_5_ nanoparticles. The addition of nanoparticles to PMMA mainly influenced the optical transmission and glass transition temperature of composite films compared to pure PMMA. It is clearly visible in the results of the conducted ellipsometric and differential scanning calorimetry tests. X-ray studies showed that the heat treatment of the samples resulted in the ordering of the polymer structure (flattening of the polymer chains). Examining the surface of the samples with scanning electron microscopy, it can be seen that Nb_2_O_5_ nanoparticles formed unusual, branched formations resembling "snowflakes".

## Introduction

In recent years, polymer composites based on inorganic oxides have aroused increasing interest in such fields of science as materials science or related fields (physics and chemistry of materials). Polymer/inorganic oxide composites often exhibit different material properties compared to pure polymers. They often differ from pure polymers in their optical, thermal and electrical properties. One of the most studied and more frequently modified polymeric materials is polymethyl methacrylate (PMMA). This commonly used thermoplastic material has good tensile strength, hardness, high rigidity and optical transparency, and is a good electrical insulator. It is also widely used in practical applications including optics, telecommunications, and even in the production of various general-purpose products. There are many works on PMMA applications in the literature, for example see reviews from the last few years^1–7^. Due to such favorable properties of PMMA, this material is also often subjected to various modifications. In the literature, it can be find many examples of PMMA modification with the use of nanoparticles and nanowires, including TiO_2_^8–11^, Ag^12–14^, ZnO^15–18^ and their mixtures, incl. the use of other types of nanoparticles^19–22^. In particular, these studies show that the addition of Al and TiO_2_ nanoparticles to the PMMA resin lowers the glass transition temperature of this material and increases its electrical conductivity^11^. The addition of nanoparticles can change the optical properties of PMMA, such as absorption. In the work of Deng et al.^13^ PMMA/Ag nanocomposites were studied. It was observed that the addition of Ag nanoparticles causes an increase in the nonlinear optical properties of the composite. Moreover, such composites are quite widely used in optical devices^13^. As the conductive ink, a PMMA/AgNW (nanowires) composite can be used, as shown by Martínez et al.^14^. The ink layers thus formed have a very low conductivity and can be widely used in soft lithography. At this point, PMMA/ZnO composites should also be mentioned. These photocatalytic composites are very stable. In the works of Di Mauro et al.^15^ PMMA/ZnO nanocomposites have been investigated as a modern photocatalyst used in water treatment. The results are presented on the example of the degradation of the dye methylene blue (MB) and phenol in aqueous solution under the influence of UV radiation. The composites retained their properties and were still stable after several decolorization processes. From these few examples it is clear that PMMA composites with nanoparticles or nanowires also have a wide range of applications.

The niobium pentoxide—Nb_2_O_5_^23–27^ is an inorganic compound used as the main precursor of all materials made of niobium. It has a specialized application in devices such as capacitors. It is also used as a component of optical glasses. A characteristic feature of the layers of niobium oxide itself is optical transmittance in almost the entire spectral range (300–2500 nm). Atta et al.^23^ show how annealing affects the structural, optical and electrical properties of Nb_2_O_5_ layers. It turned out that in the range of temperatures up to 200 °C the transmission level, the values of the refractive index and absorption were stable.

Here, we have provided variable-temperature spectroscopic ellipsometry and differential scanning calorimetry studies of glass transition of PMMA/Nb_2_O_5_ composite films. There are some literature data, where PMMA Tg was determined using raw ellipsometric data, e.g. ellispometric angles—Ψ and Δ^28–32^. In the work of Erber et al.^32^ PMMA films, deposited onto Si/SiOx substrates were investigated. The glass transition temperature was determined using Ψ and Δ temperature relations, at λ = 450 nm wavelength. This example shows that Tg of pure PMMA is around 107 °C. Another example is work of Keddie et al.^31^, where Tg was determined using temperature derivative of ellipsometric angle delta, at λ = 232 nm wavelength. Similarly to work^32^, the glass transition temperature was around 107 °C. Here, we have also used raw ellipsometic data (the Ψ angle at λ = 900 nm wavelength) for determination of Tg of obtained films, like in our earlier works^33–35^.

The aim of the work was to prepare PMMA/Nb_2_O_5_ composite films, the transmission of which in the spectral range of UV radiation (250–380 nm) is clearly reduced. And also showing the differences in physical properties between PMMA and composite PMMA/Nb_2_O_5_ films depending on their composition. It seems that the addition of a small amount of nanoparticles resulted in different thermal, physical and morphological properties of the composite film. On the other hand, Nb_2_O_5_ nanoparticles in composite films formed branched structures resembling snowflakes on a microscopic scale.

## Experimental

**Figure 1:**
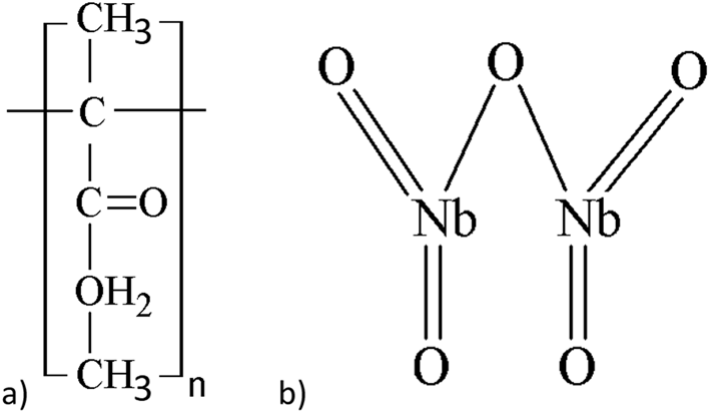
Chemical structures of PMMA (**a**) and Nb_2_O_5_ (**b**).

The materials we have used are poly(methyl methacrylate)—PMMA (molar mass 84,000 g/mol) and niobium pentoxide Nb_2_O_5_ (purity 99.9%). PMMA and Nb_2_O_5_ were supplied by Sigma-Aldrich. The chemical structures of these materials are shown in Fig. 1.

PMMA was dissolved in chloroform. The weight concentrations of all solutions were constant and equal to 20 mg/ml. The percentage content of Nb_2_O_5_ in relation to the PMMA and weight of individual contents are presented in Table 1.

**Table 1:** Weight and concentration of individual chloroform/polymer/NPs solutions.

NPs content (%)	0	2.5	5	10	20
PMMA weight (mg)	20	19.5	19	18	16
Nb_2_O_5_ Weight (mg)	0	0.5	1	2	4

All solutions were homogenized at 16 kJ for 10 min using a Bandelin Sonoplus homogenizer. From these solutions, films of PMMA and PMMA/Nb_2_O_5_ composites were casted onto silicon substrates (coated with 400 nm thick SiO_2_ layers) and onto opaque and quartz substrates. Samples were annealed at 180 °C, for 5 min. All measurements, with exception of temperature-dependent ellipsometry and DSC were taken before and after annealing.

The films intended for ellipsometric measurements were annealed at the beginning of the procedure of the glass transition temperature determination, what is described in further part of this section.

All prepared films were stored in a dry laboratory box at room temperature. The dry box, with a rubber gasket, was half filled with hygroscopic gel and closed under nitrogen atmosphere, in a glove box.

The ellipsometric measurements were made using the SENTECH SE850E spectroscopic ellipsometer, which operates in the spectral range of 240–2500 nm and works under the control of the Spectra Ray 3 software. The device operates in three modes: transmission mode (using a special sample holder), variable angle mode (using a standard automatic table) and variable temperature mode (using a variable temperature cell operating at reduced pressures and under temperature controller INSTEC mK1000). The measurements in transmission mode were performed in all UV–Vis/NIR spectral range. The measurements of ellipsometric angles Ψ and Δ were performed for incidence angle 40°–70° range with the 5° step. The variable temperature measurements were provided in accordance with the protocol described in our previous works^33–35^. Every, individual samples were heated at 180 °C, during 5 min, under pressure (to 10^−1^ Tor). Next, the films were quickly cooled to − 20 °C during 3 min time. The temperature has been set by the temperature controller using a liquid nitrogen pump and electric heater. The transmission mode was used for optical transmission measurement, the variable angle mode for thickness determination and variable-temperature mode for T_g_ determination.

The glass transition temperatures of samples were also determined using a DSC Q2000 apparatus (TA Instruments, Newcastle, DE, USA), with aluminium sample pans. Thermal characteristics of the samples were obtained under nitrogen atmosphere (gas flow 50 mL/min). The instrument was calibrated with high-purity indium standards. DSC measurements have been performed on powder materials obtained from very thick films (about 2 μm), which were removed from glass substrates. All films, intended for DSC measurements were annealed earlier^36^.

X-Ray diffraction studies were performed using the D8 Advance diffractometer (Bruker, Karlsruhe, Germany) with Cu-Kα cathode (λ = 1.54 Å). Due to relatively high layer thickness of sample (~ 1000 nm), for 2D-WAXS setup the classic Bragg–Brentano geometry measurement was applied. The scan rate was 1.2°/min with scanning step 0.02° in range of 2° to 60° 2Θ (dwell time 1 s). Measurements were taken in 2 variations, using different φ (Phi) angle, what corresponds to sample rotation. As a φ = 0°, longer edge was set as parallel to X-Ray beam direction. Resulting φ rotation (90°) were programmed with resolution of 0.1° φ. Obtained 2D patterns (with width of 3° 2θ) for different φ angle were integrated to 1D patterns. Background subtraction, occurring from air scattering, were performed using DIFFRAC.EVA program^36^. All WAXD (XRD) measurements acquired at different Phi angle were accumulated to obtain representative pattern. The Nb_2_O_5_ parameters, determined using X-ray diffraction are shown in Table 2.

**Table 2:** Nb_2_O_5_ parameters determined using X-ray diffraction.

Powder	Space group	Lattice parameters, ICDD, Å	Lattice parameters, calculated, Å	Crystallite size, nm	Lattice strain, %
Nb_2_O_5_ (00–027-1003)	Pbam	a = 6.168, b = 29.312, c = 3.936	a = 6.17, b = 29.31, c = 3.93	65 ± 3	− 0.42 ± 0.03

SEM images were obtained using the Zeiss Supra 35 scanning electron microscope, which accelerating voltage is in 2–4 kV range, work distance in 3–3.5 range. The images were collected using InLens mode (for flat and nanometric samples).

The molar mass was determined using Gel permeation chromatography with multiangle laser light scattering detection (GPC-MALLS). Analysis was performed in THF at 35 °C with a nominal flow rate of 1 mL/min. A column set containing SDV columns from Polymer Standards Service (PSS, Mainz, Germany): guard + 100 Å + 500 Å + 1000 Å + 100,000 Å was used. A differential refractive index detector (Δn-2010 RI WGE Dr. Bures, Berlin, Germany) and a multiangle laser light scattering detector (DAWN HELEOS from Wyatt Technologies, Santa Barbara, USA) were used in the system. The results were evaluated with ASTRA 5 software (Wyatt Technologies, Santa Barbara, USA).

## Results and discussion

**Figure 2:**
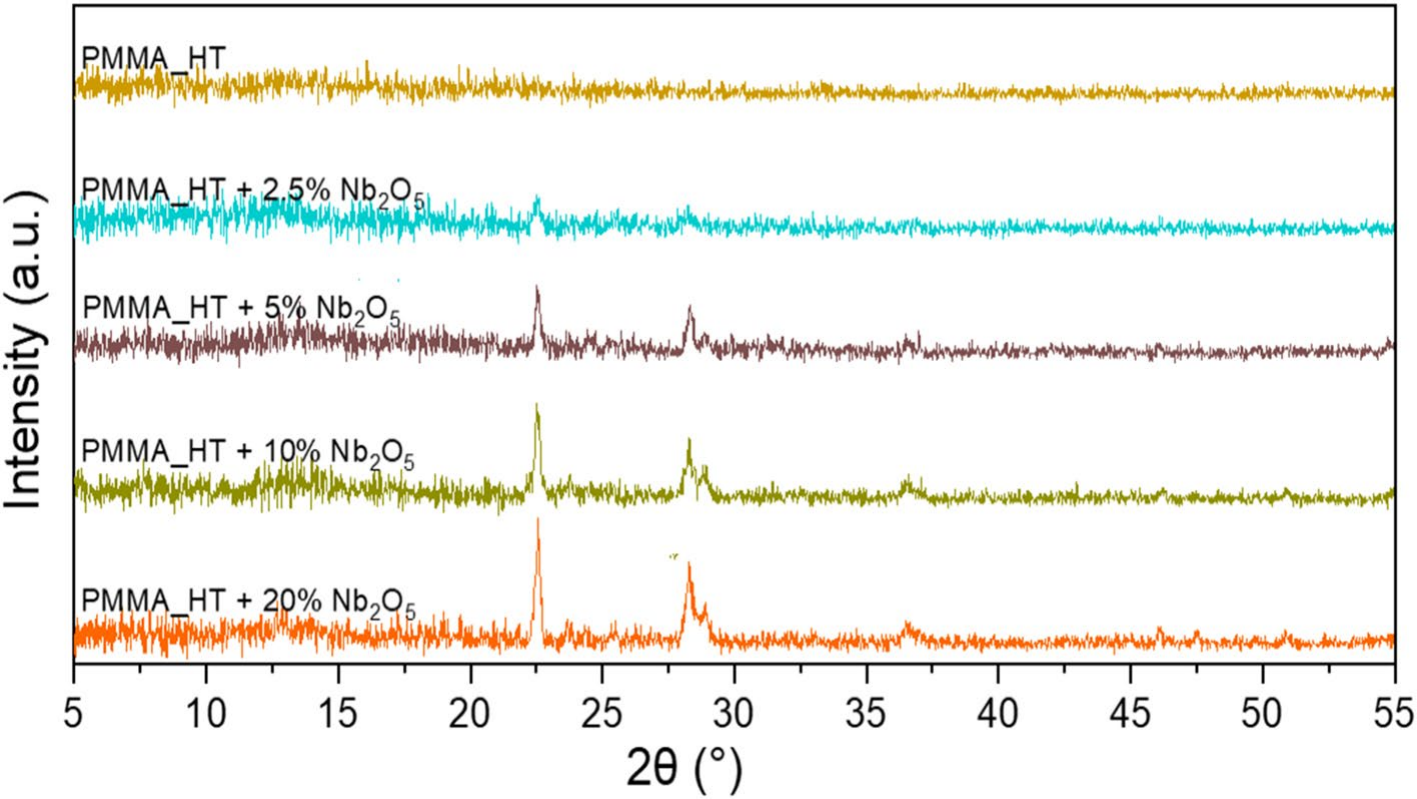
XRD patterns of PMMA films with increasing content of Nb_2_O_5_.

The X-ray diffraction patterns performed on annealed films deposited onto Si substrates are shown in Fig. 2. The diffraction patterns of no-annealed films are show in supplementary materials in Fig. 1s. The diffraction pattern of Nb_2_O_5_ nanoparticles powder has been added in supplementary also. The pure PMMA spectrum is shown in yellow colour, as the upper curve in the figure. The next curves come from samples with the addition of Nb_2_O_5_. The "HT" curves represent the films subjected to a temperature of 180 °C. The spectrum of pure PMMA is completely free of peaks in opposite to spectrum of no-annealed film (in supplementary), where only amorphous background is visible. This means that the polymer chains have flattened and the entire structure of the film has become more orderly. The same effect has been observed in spectra of the remaining, annealed samples, where only peaks originating from Nb_2_O_5_ are visible. It can also be noticed that the spectra of the samples with a higher Nb_2_O_5_ content have a higher intensity of these peaks and a larger number of them, which is consistent with the preparation of the obtained films.

**Figure 3:**
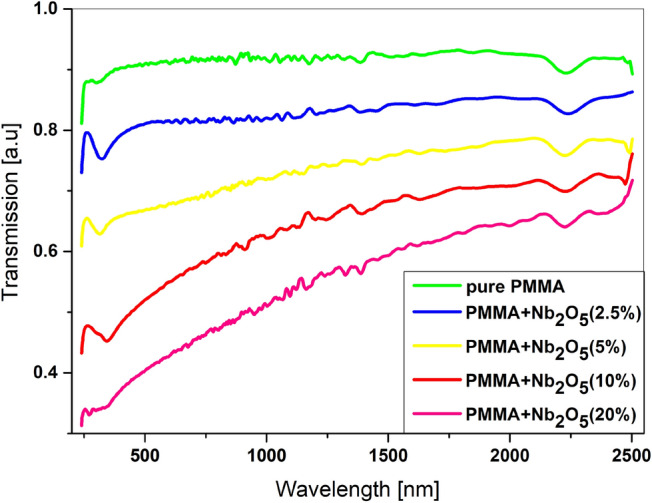
Transmission of pure PMMA and PMMA/Nb_2_O_5_ films.

The transmission spectra of annealed, pure PMMA and PMMA/Nb_2_O_5_ composites films deposited onto quartz substrates are shown in Fig. 3. The transmission intensities of pure PMMA before annealing (see Fig. 3s in supplementary) and after thermal treatment are about 92%. The change in the shape of the spectrum in the UV range after annealing suggests the ordering of the polymer structure. A similar effect was observed for the annealed layers of PMMA/Nb_2_O_5_ composites. It has been observed that a higher percentage of nanoparticles causes a decrease of light transmission in the UV range. In the remaining range, the permeability remains at the level of 60%, even in case of the maximum of nanoparticles content.

**Figure 4:**
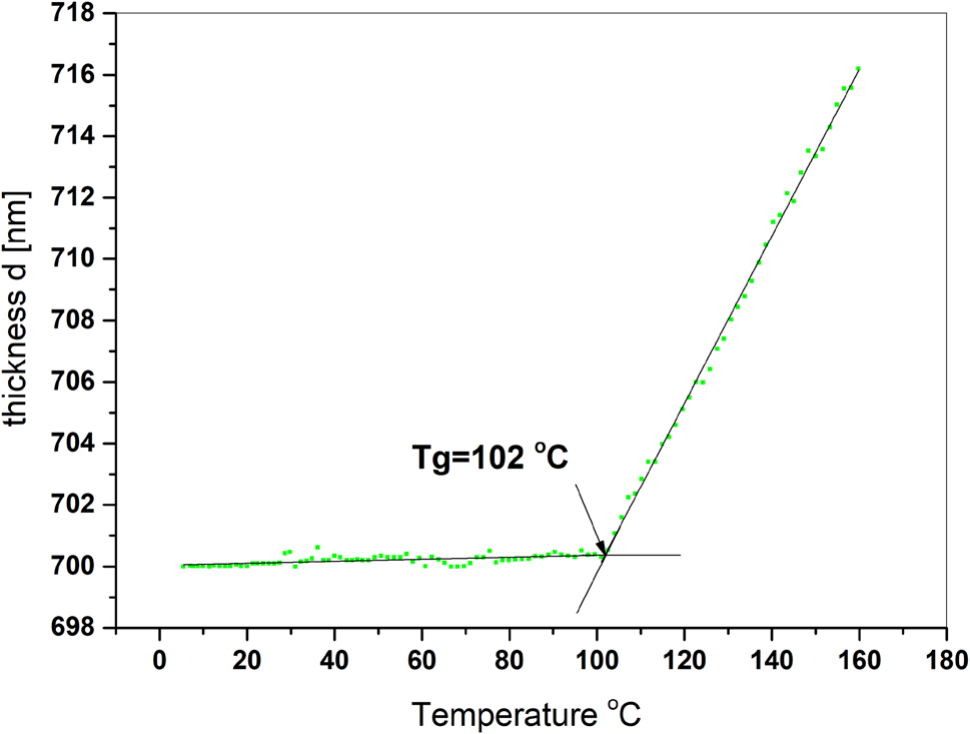
Thickness of pure PMMA as temperature function.

In Fig. 4, the thickness of pure PMMA as a function of temperature is presented. It can be readily observed from Fig. 4, that the value of thickness is increasing with temperature and the glass transition is around 102 °C. The mean square error (MSE) of this fitting was around 0.195. Dependence of thickness on temperature was determined ellipsometrically. The ellipsometric angles spectra of investigated samples were fitted with Cauchy optical model in 830–930 nm wavelength range, similarly, like in our earlier works^33–35^. The Cauchy optical model parametrizes the dependence of the refractive index n and the absorption (extinction) coefficient k on the wavelength λ and is described by following relation:

$$n(\lambda ,T)={n}_{0}(T)+{C}_{0}\frac{{n}_{1}(T)}{{\lambda }^{2}}+{C}_{1}\frac{{n}_{2}(T)}{{\lambda }^{4}},$$

$$k(\lambda ,T)={k}_{0}(T)+{C}_{0}\frac{{k}_{1}(T)}{{\lambda }^{2}}+{C}_{1}\frac{{k}_{2}(T)}{{\lambda }^{2}},$$

where C_0_ and C_1_ are numerical constants, k and n are already mentioned coefficients.

The ellipsometrically determined thicknesses of samples deposited on silicon substrates are presented in Table 3.

**Table 3:** Thickness of films, deposited on different substrates.

NPs content (%)	0	2.5	5	10	20
Thickness d (nm) of films onto silicon substrates for XRD	703	727	669	693	650
Films on silicon substrates for ellispometry	844	523	651	835	842
Films on quartz substrates for transmission	384	420	430	443	450

The correlations between temperature dependences of thickness and ellipsometric angles were presented in earlier works^33–35^. The raw ellipsometric data are used for glass transition determination quite frequently, what can be found in literature^37–45^.

**Figure 5:**
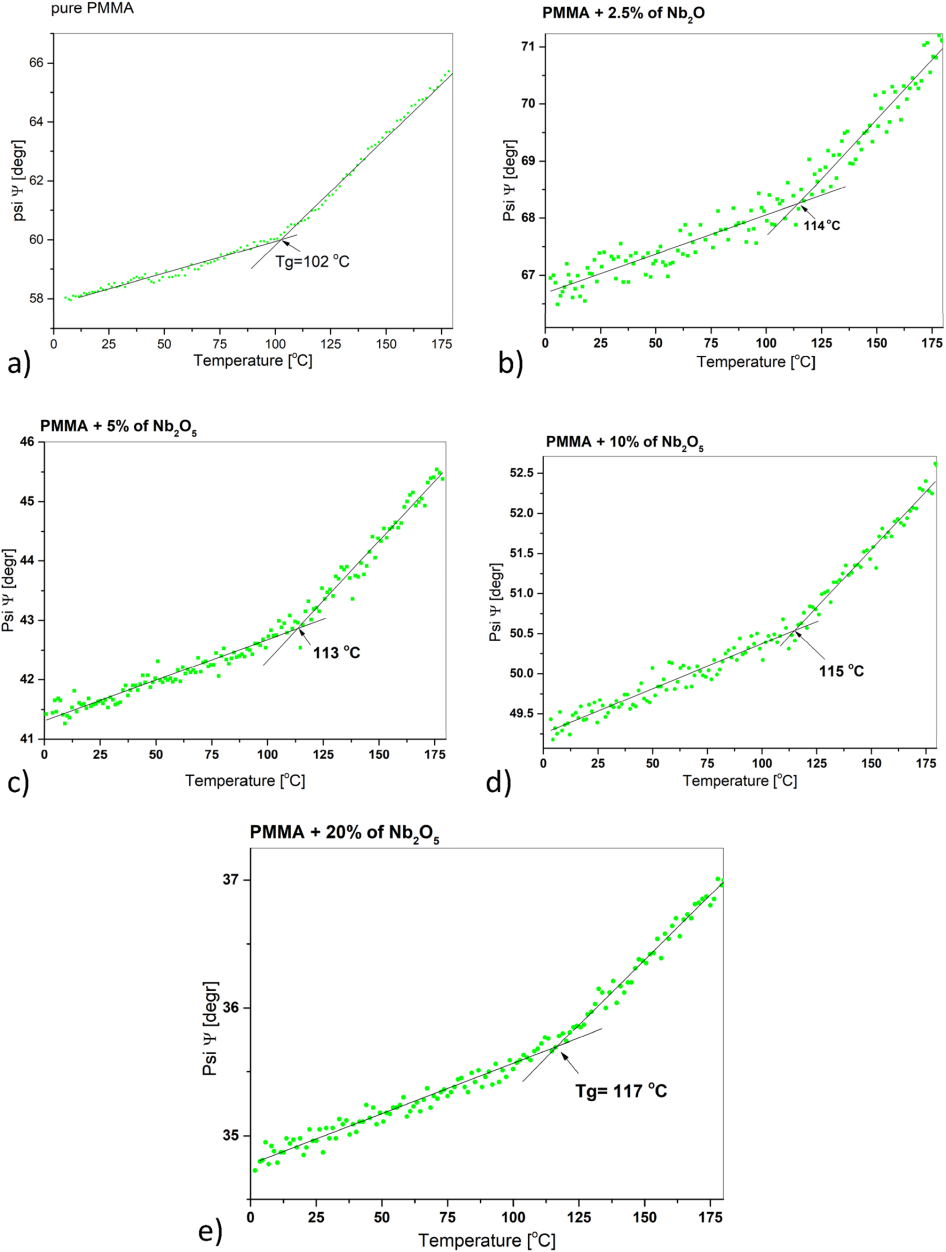
Ellipsometric angle Ψ at 900 nm as a function of temperature for: (**a**) pure PMMA, (**b**) PMMA/Nb_2_O_5_ (2.5%), (**c**) PMMA/Nb_2_O_5_ (5%), (**d**) PMMA/Nb_2_O_5_ (10%), (**e**) PMMA/Nb_2_O_5_ (20%).

Here, we have chosen the Ψ at λ = 900 nm wavelength. The reason for choosing this wavelength is the lack of distinct absorption bands in this range, and the limited spectral range in temperature measurement. The spectra were collected only in the range of 240–930 nm due to the time scale of the measurement (15 s), the measurement frequency (one measurement every 10 s) and the heating rate (2 °C/min). Measurement in the entire spectral range takes at least 2 min and therefore it is not reliable in variable temperature measurements carried out in-situ. In Fig. 5, the Ψ angle as a function of the temperature is shown for the pure PMMA and PMMA/Nb_2_O_5_ composite films. The glass transition of pure PMMA is equal to 102 °C, while the glass transition of all composite films is higher on average by 10 °C. The glass transition of PMMA/Nb_2_O_5_ with 2.5% concentration, is around 114 °C. Glass transition temperatures of successive of composite films, with concentrations of 5, 10 and 20% are around 113, 115 and 117 °C. The linear fittings for presented curves were performed using the least squares method. The fitting parameters, including standard deviation errors, are attached to supplementary materials in point 4 (Figs. 9–14s and Table 1s).

It can be easy seen that dispersion of collected points is slightly larger for Ψ(T) relation in PMMA/Nb_2_O_5_ (2.5%). This is result of thickness effect, which has been already described in literature^46^.

Along with the increase of the Nb_2_O_5_ nanoparticles concentration in the composite, there is a slight upward trend in the film glass transition temperature.

**Figure 6:**
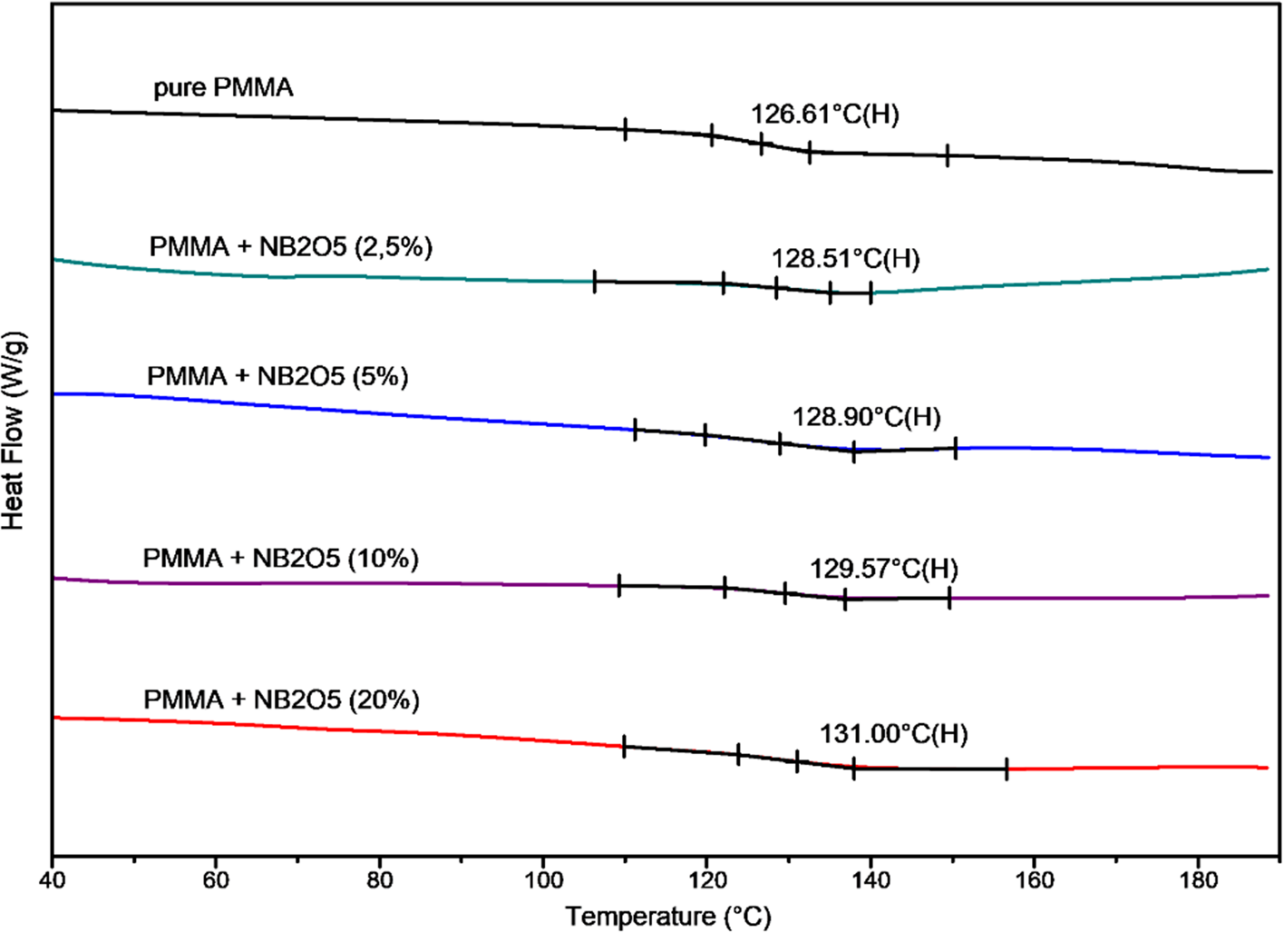
DSC plots, with a heating rate of 20 °C/min, for pure PMMA, and PMMA/Nb_2_O_5_ (2.5, 5, 10, and 20%).

The obtained dependence is fully consistent with the results of the DSC measurement, which are shown in Fig. 6. We have performed DSC studies on materials, removed from opaque glass substrates (the samples were annealed at 180 °C at first). The calorimetric measurements Nb_2_O_5_ were performed with constant heating rate of 20 °C/min. As in the case of the ellipsometric results, a slight increase in the glass transition temperature of the composite can be noticed along with the increasing concentration of Nb_2_O_5_. The glass transition of pure PMMA is around 126 °C, while the glass transition temperatures of samples with concentrations of 2.5, 5, 10 and 20% nanoparticles are around 128.5, 128.9, 129.6 and 131 °C, respectively. The difference between values obtained from ellipsometry and from DSC results from the heating rate, which was 2 °C/min and 20 °C/min respectively. The difference also results from the form of materials.

**Figure 7:**
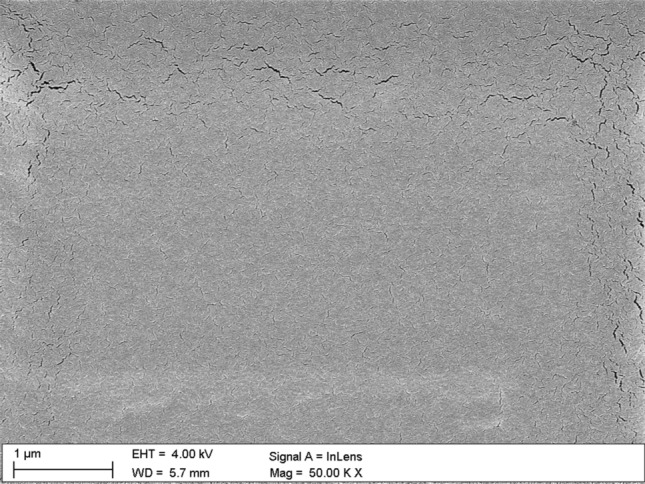
Surface of PMMA film.

**Figure 8:**
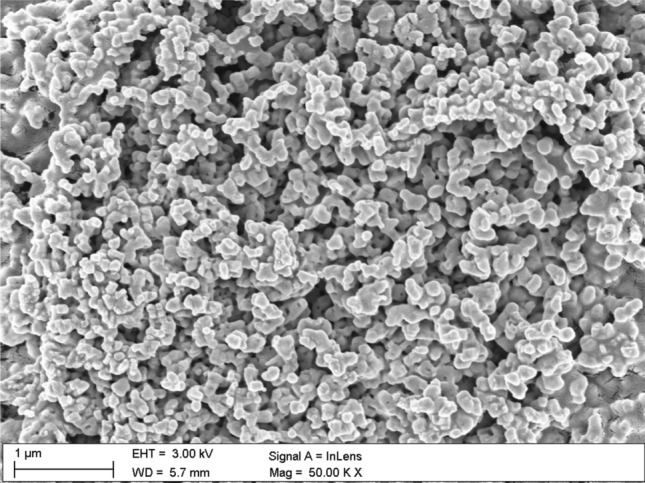
The Nb_2_O_5_ nanoparticles clusters in PMMA film.

**Figure 9:**
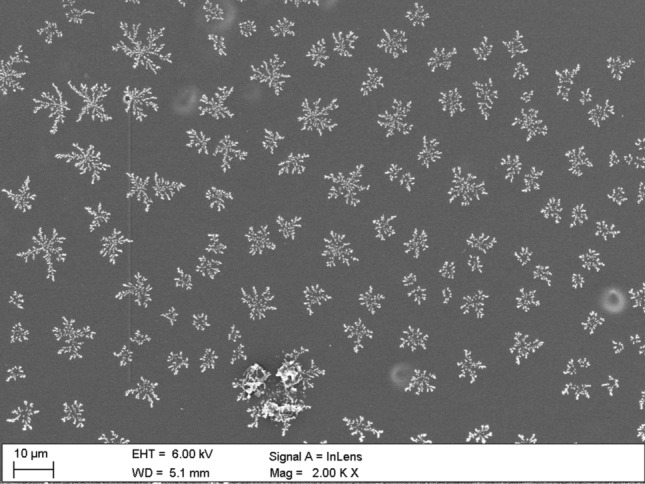
The morphology of PMMA/Nb_2_O_5_ composite film in macroscopic scale.

Figures 7, 8, 9, 11 and 12 show the images performed using scanning electron microscope. The surface of pure PMMA annealed film is shown in Fig. 7 and the surface of no-annealed one is presented in supporting materials (Fig. 15s).

The example cluster of Nb_2_O_5_ nanoparticles, after annealing, magnified 50.000 times, is shown in Fig. 8, the no-annealed one is attached in supporting materials (Fig. 16s).

Surface of the annealed PMMA polymer films seems to be slightly cracked in relation to the no-annealed one. These visible "microcracks" are the result of local burning out of the polymer surface by an electron beam. The melting point of the used PMMA is about 180 °C and all the films were annealed to this characteristic temperature value. The melting point of Nb_2_O_5_ nanoparticles is at about 1512 °C. Therefore, the annealing temperature of composite films did not affected the formations of nanoparticles in composite films. An examples of Nb_2_O_5_ crystallization forms can be found in the literature e.g.^47–52^. The Nb_2_O_5_ formations can appear in spheroidal form, the so-called hollow spheres^47–49^. A good example is the work of Kong et al.^47^, where hollow spheres were created by a hydrothermal method, in a few steps. The first stage of post-treatment was calcination in air stream, at 600 °C, the second stage was heat treatment in nitrogen N_2_, at 800 °C, and the third stage was air oxidation at 300 °C. The diameter of obtained hollow spheres was around 500 nm to 3 µm. Another type of structures that can be formed by Nb_2_O_5_ nanoparticles are the so-called nanorods^50^, microcones^51^, and hexagrammoids^52^.

**Figure 10:**
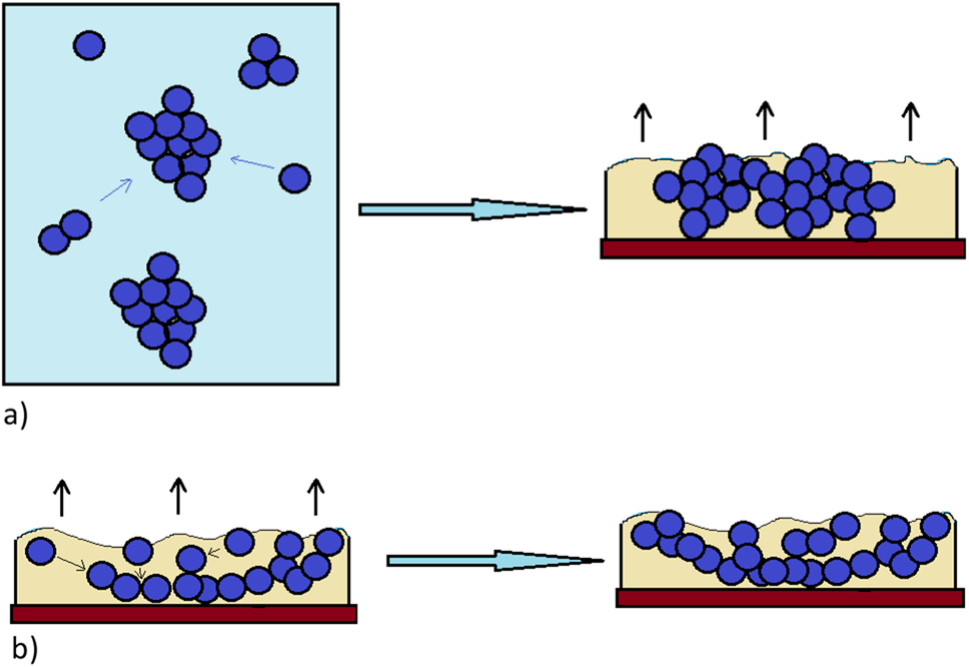
(**a**) Formation of clusters: the nanoparticles, which have aggregated in conglomerates in solution, are forming bigger clusters during casting the solution and evaporation of solvent. (**b**) Snowflake formation: individual nanoparticles and nanoparticles in small conglomerates aggregate during solvent evaporation.

“Snowflakes”-shaped branched structures in Fig. 9 are presented in macroscopic scale. This formations are visible on the surface of the annealed PMMA/Nb_2_O_5_ composite film. We suppose these structures were created as a result of two processes—adhesive interactions between nanoparticles and rapid evaporation of the solvent. The adhesive effect is responsible for the formation of clusters of nanoparticles, presented in Figs. 18s–19s, in supplementary. The “snowflakes” are formed by nanoparticles aggregation during evaporation of chloroform and polymer congelation. Besides of branched structures formation, there are also typical nanoparticle clusters, which has been started to aggregate earlier, even before the solvent has been evaporated. There is no possibility to eliminate the clusters completely. The mechanisms of clusters and snowflakes formations are presented in Fig. 10a,b.

The surface morphology is due to formation of snowflake-shaped nanoparticle clusters. Sometimes, evaporation of solvent from the layer is connected with coffe-ring effect^53,54^, but here the formation of snowflakes is rather close to nanoparticles branching effect, known in literature^55,56^.

**Figure 11:**
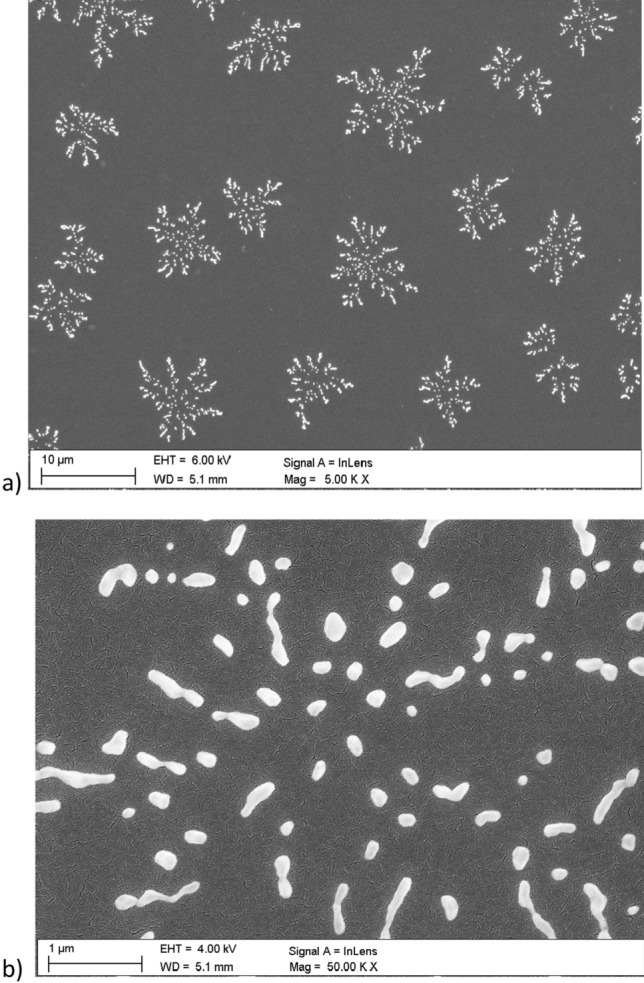
The Nb_2_O_5_ “snowflakes” in surface of PMMA/ Nb_2_O_5_ composite film with 2.5% nanoparticles content (**a**). The single “snowflake” (**b**).

**Figure 12:**
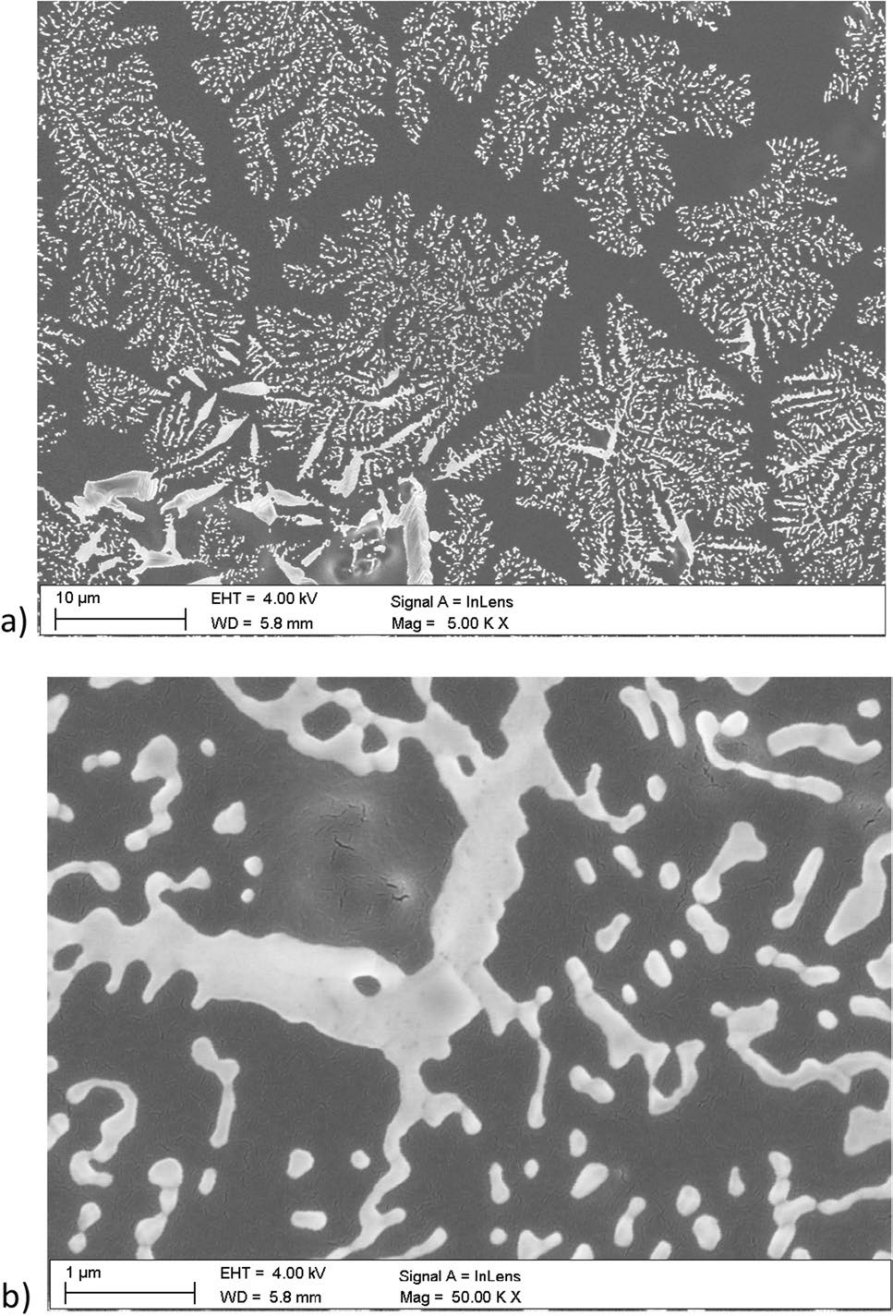
The Nb_2_O_5_ “snowflakes” in surface of PMMA/Nb_2_O_5_ composite film with 10% nanoparticles content (**a**). The fragment of “snowflakes” (**b**).

Figures 11 and 12 present the surface of selected, annealed PMMA/Nb_2_O_5_ composite films. There is surface of PMMA/Nb_2_O_5_ composites with contain of 2.5%—Fig. 11a,b and 10% nanoparticles—Fig. 12a,b. The size of “snowflakes” structures, visible in Fig. 12a is in range 10–30 µm. It is a result of higher Nb_2_O_5_ weight concentration.

The branched structures strongly indicate the formation of a polymer/NPs composite, where Nb_2_O_5_ nanoparticles cross-linked into branched formations inside the investigated film. The "snowflakes", which are visible on the polymer surface are only the top of the cross-linked structures, inside the polymer.

## Conclusions

In presented work, pure PMMA and PMMA/Nb_2_O_5_ composite films were investigated. Transmission, variable-angle and variable temperature spectroscopic ellipsometry, X-ray diffractometry, DSC and scanning electron microscopy were used for the analysis of the obtained samples. It was observed that the addition of a small amount of Nb_2_O_5_ nanoparticles causes the composite films to differ from pure PMMA in terms of optical and thermal properties, as well as clear changes in their morphology. Due to the higher concentration of nanoparticles, the optical transmission intensities of the investigated samples were lower, mainly in the UV wavelength region. The reduced transmission in this range means that the described composite may have potential use in optical devices. Annealing of the tested samples increased the ordering of the entire layer structure, which was confirmed by X-ray diffraction measurements. Additionally, the higher concentration of nanoparticles caused the T_g_ to shift towards higher temperature values. This was confirmed by elispometric and calorimetric tests. Snowflake-shaped branched structures visible on the surface of composite films were probably the result of two processes—rapid evaporation of the solvent and adhesion of nanoparticles, i.e. their aggregation. The process of the formation of branched structures requires the optimization of technological parameters, like time and energy of homogenization and is the basis for further research.

## Supplementary Information

Supplementary Information.
