# Supplementary material for: Thermal and optical properties of PMMA films reinforced with Nb2O5 nanoparticles
Source: Sci Rep. 2021 Nov 18;11:22531. doi: 10.1038/s41598-021-01282-7 (PMC8602437; doi:10.1038/s41598-021-01282-7)
Supplement: Supplementary file 1 — Supplementary Information. [file 41598_2021_1282_MOESM1_ESM.docx]

Thermal and optical properties of PMMA films reinforced with Nb**_2_**O**_5_** nanoparticles - SUPPLEMENTARY

B. Hajduk^a,*^, H. Bednarski^a^, P. Jarka^b,*^, H. Janeczek^a^, M. Godzierz^a^, T. Tański^b^

**1. XRD diffraction patterns**

Diffraction patterns of pure PMMA and PMMA/Nb_2_O_5_ films are shown in Fig. 1s


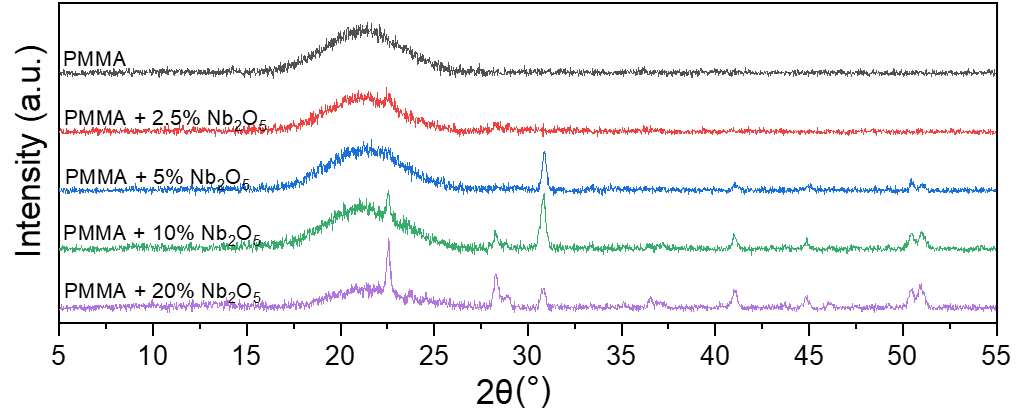


Fig. 1s Diffraction patterns of no-annealed films of PMMA and PMMA/Nb_2_O_5_

Nb_2_O_5_ diffraction pattern is shown in Fig. 2s

Fig. 2s Nb_2_O_5_ diffraction pattern

**2. Transmission spectra**

The spectra of no-annealed films of pure PMMA and PMMA/Nb_2_O_5_ composites are shown in Fig.3s


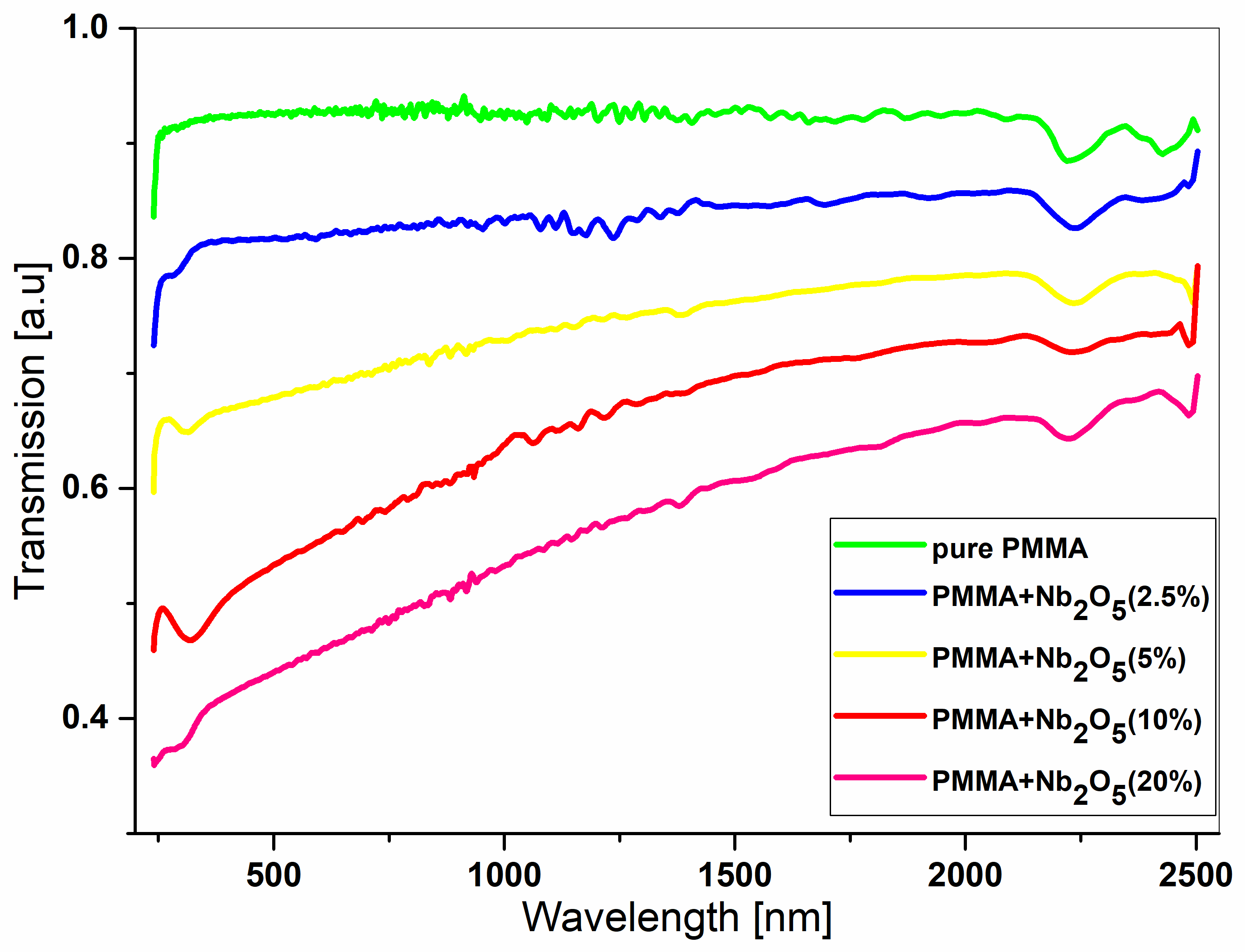


Fig. 3s Transmission spectra of no-annealed pure PMMA and PMMA/Nb_2_O_5_ composites films, deposited onto quartz substrates

**3. Ellipsometry**

The Ψ and Δ on wavelength relations, for different temperatures, collected during temperature measurements are presented in Figs. 4s-8s


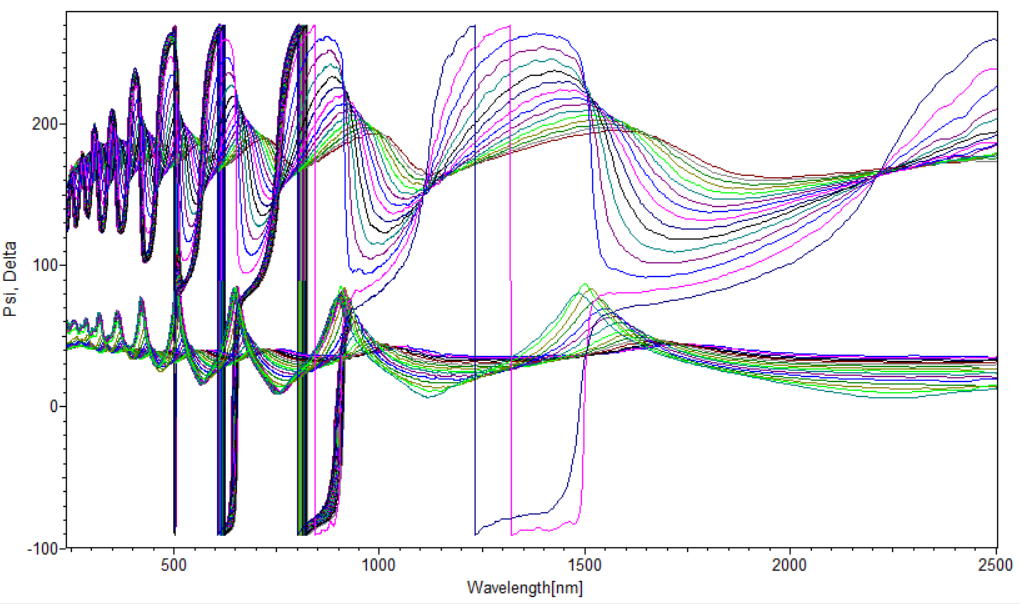


Fig. 4s Ψ and Δ temperature changes of pure PMMA film


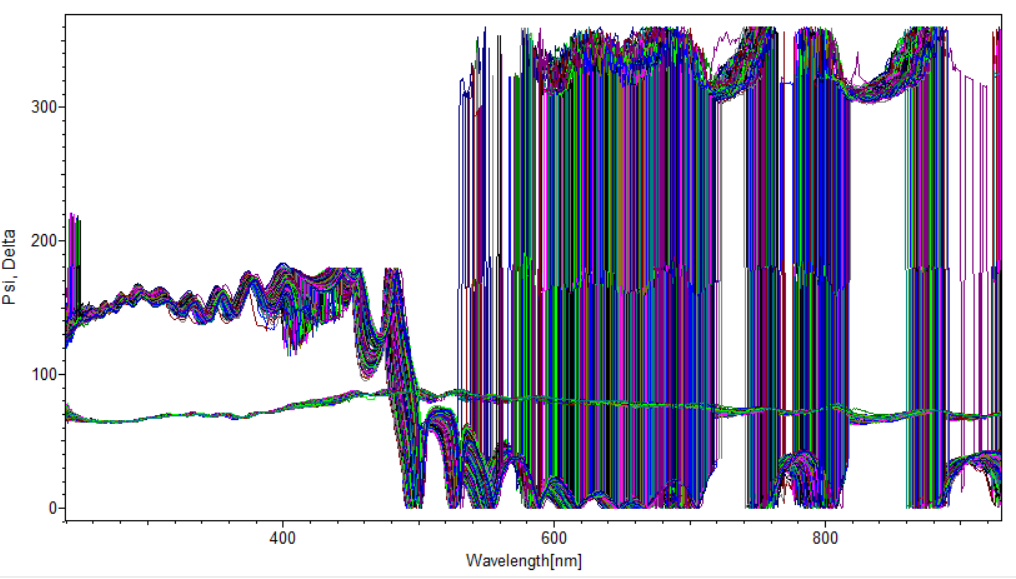


Fig. 5s Ψ and Δ temperature changes of PMMA/Nb_2_O_5_ composite film (2.5%)


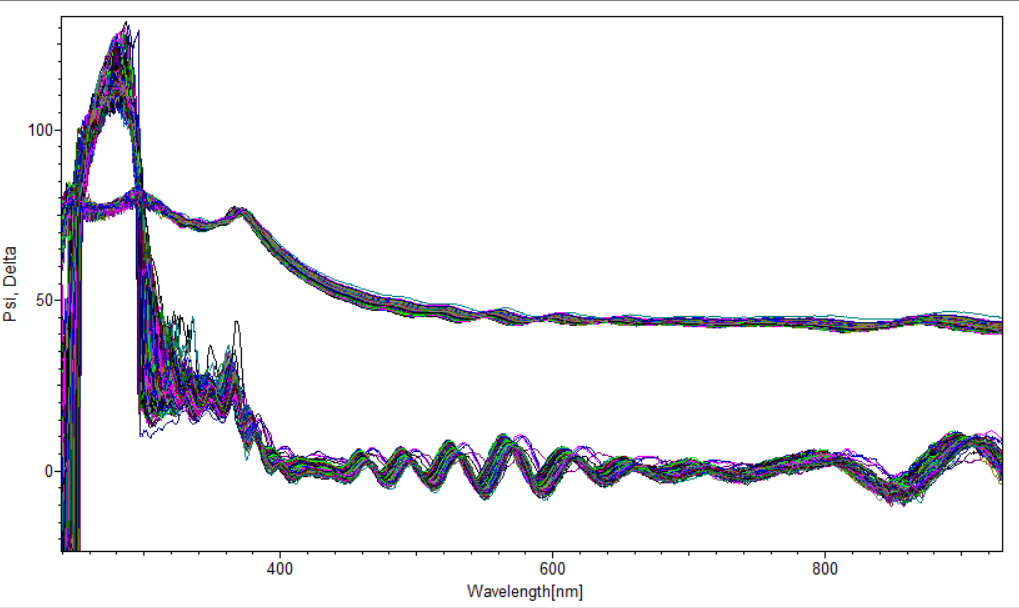


Fig. 6s Ψ and Δ temperature changes of PMMA/Nb_2_O_5_ composite film (5%)


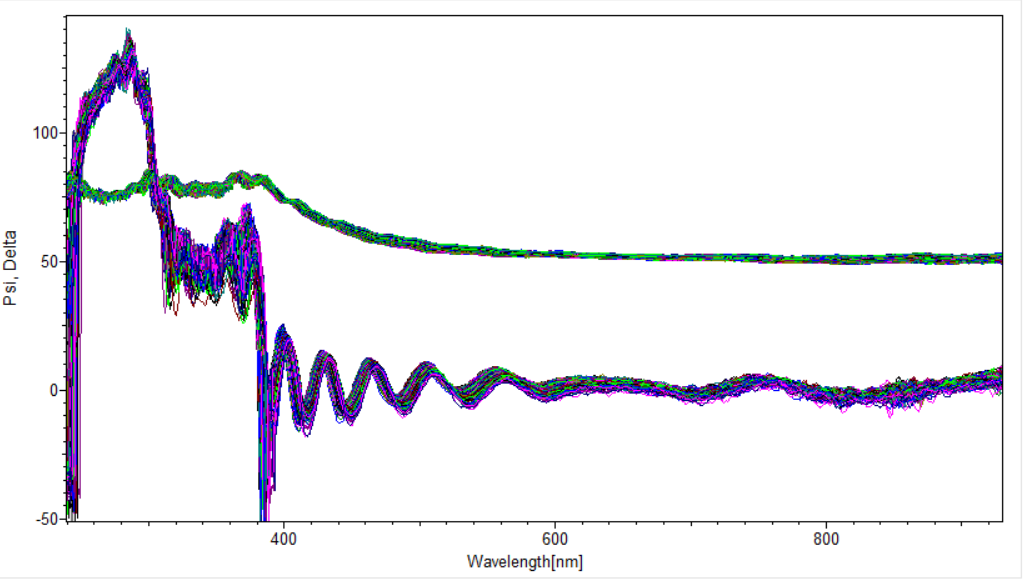


Fig. 7s Ψ and Δ temperature changes of PMMA/Nb_2_O_5_ composite film (10%)


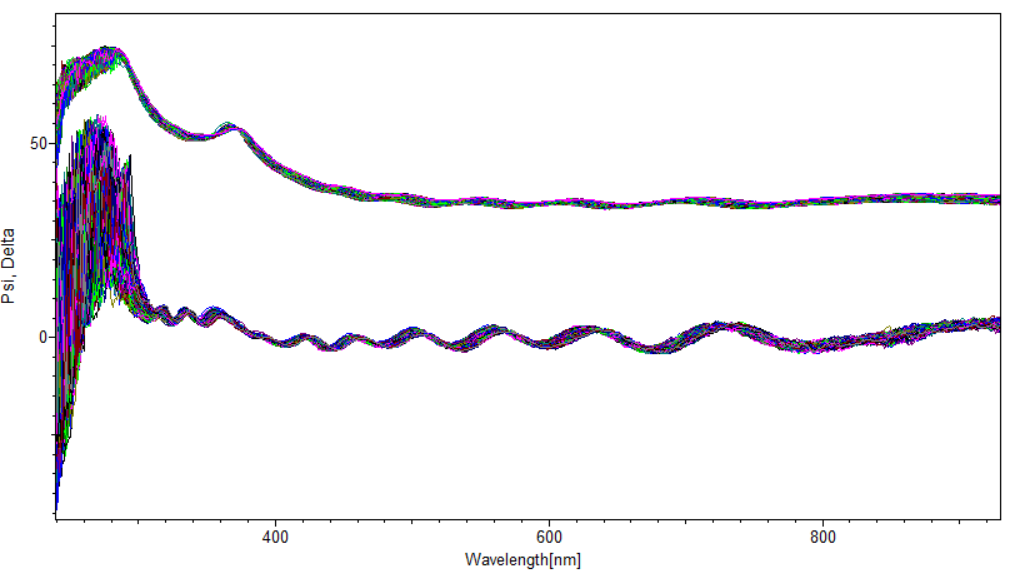


Fig. 8s Ψ and Δ temperature changes of PMMA/Nb_2_O_5_ composite film (20%)

**4. Linear fit**

Linear fits for the tested samples are presented in Figs. 9s-14s


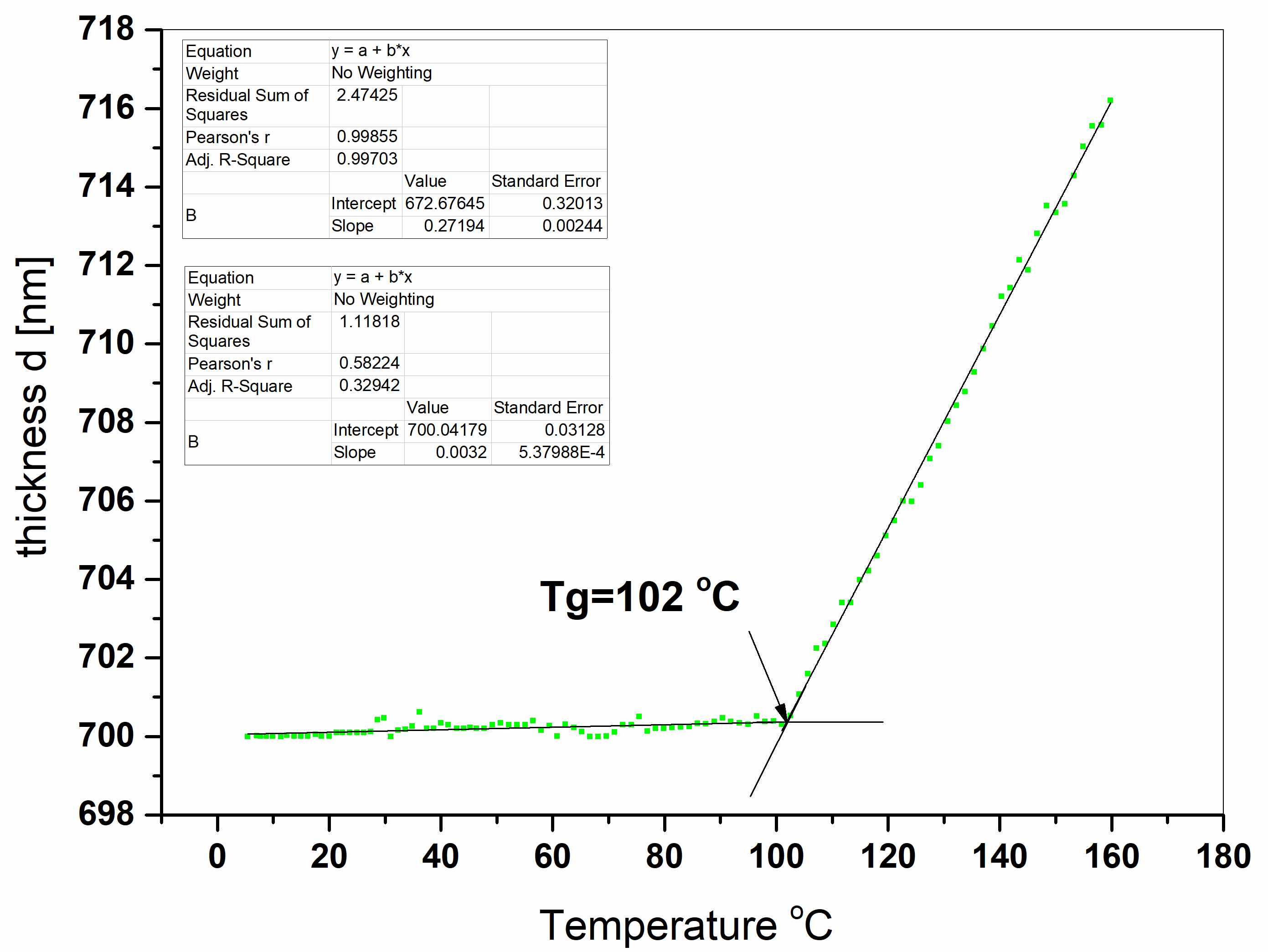


Fig. 9s Thickness linear regressions of PMMA film as a function of temperature


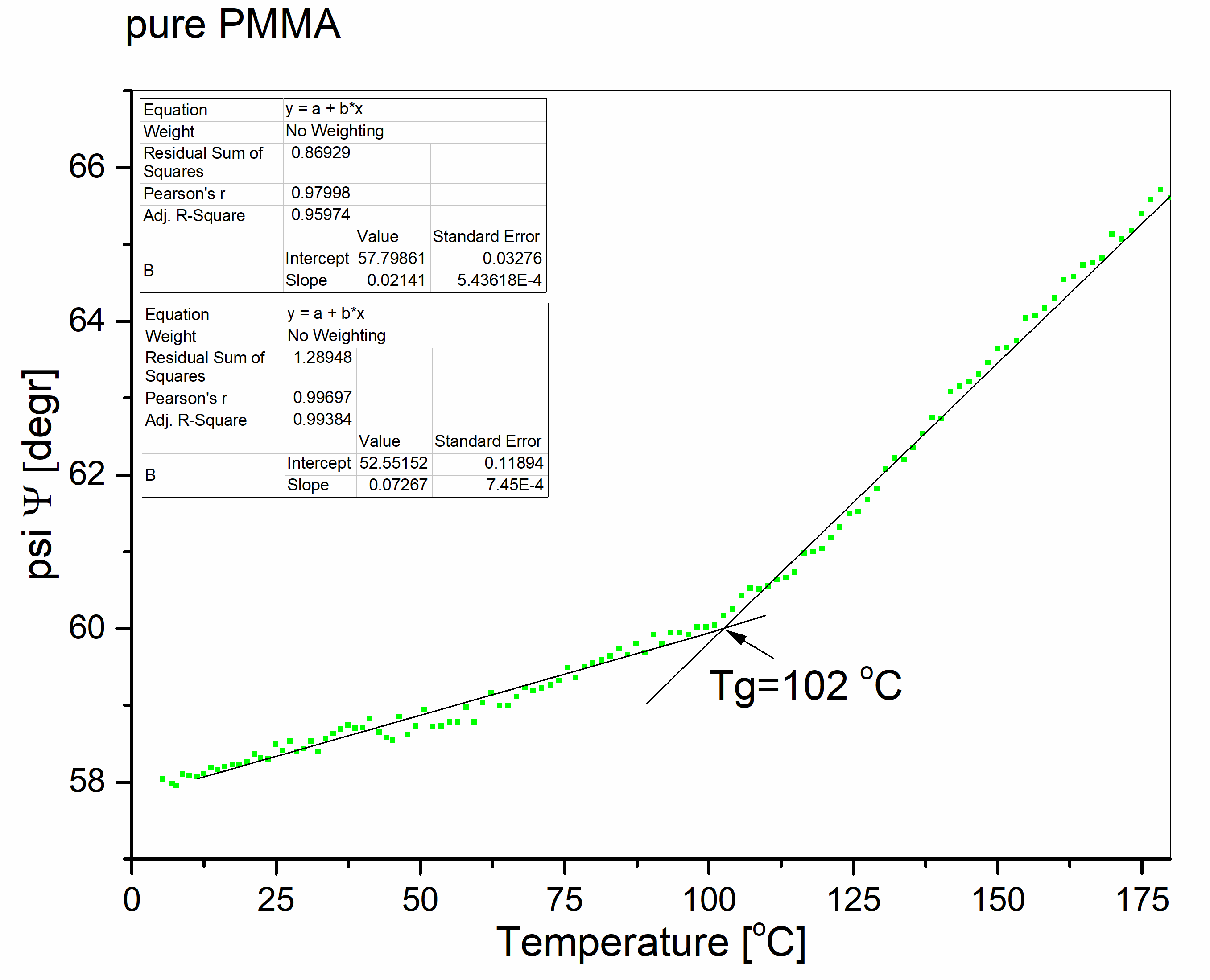


Fig. 10s Linear regressions of the ellipsometric angle Ψ as a function of temperature for the pure PMMA film


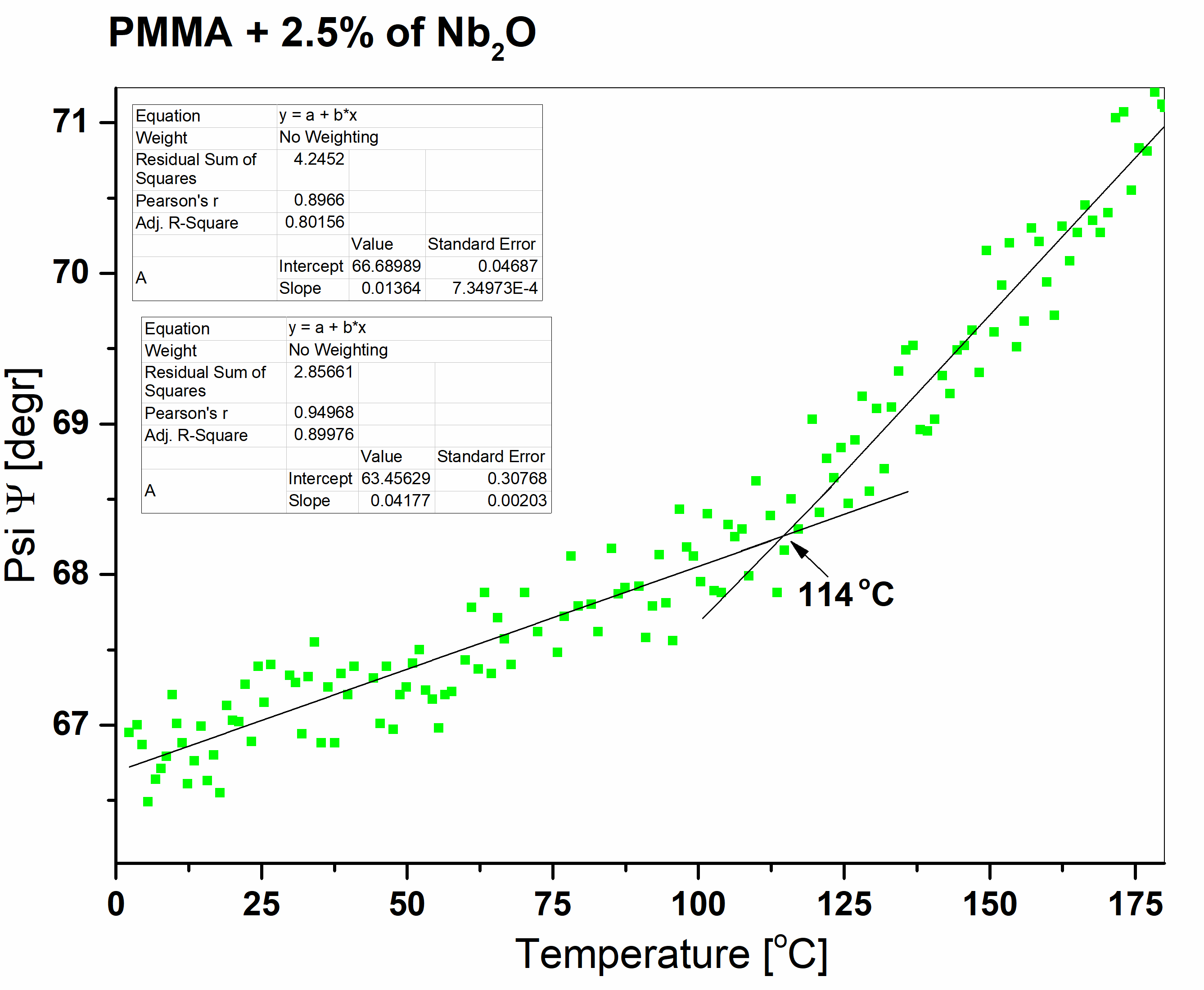


Fig. 11s Linear regressions of the ellipsometric angle Ψ as a function of temperature for the composite film PMMA/Nb_2_O_5_ (2.5%)


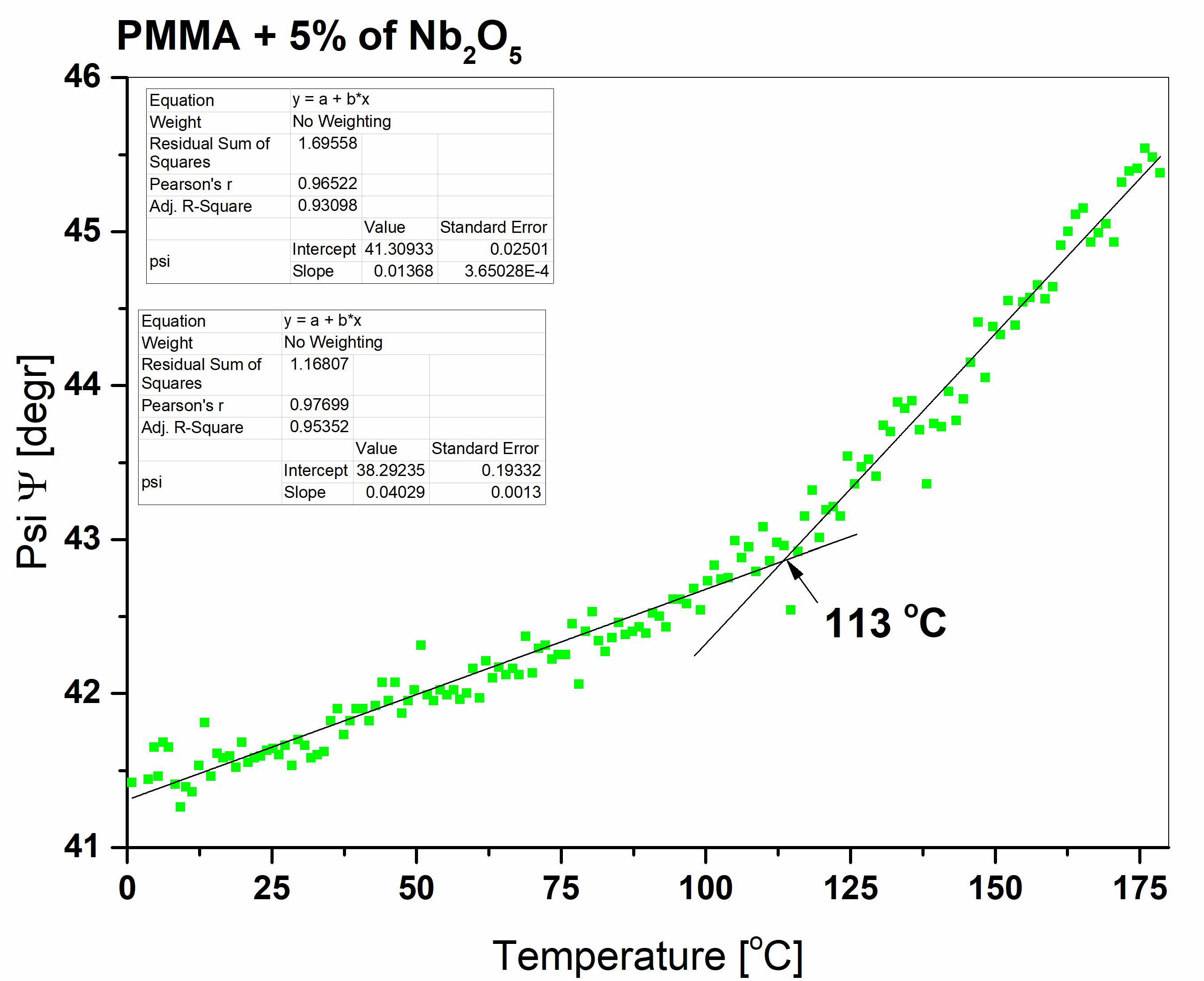


Fig. 12s Linear regressions of the ellipsometric angle Ψ as a function of temperature for the composite film PMMA/Nb_2_O_5_ (5%)


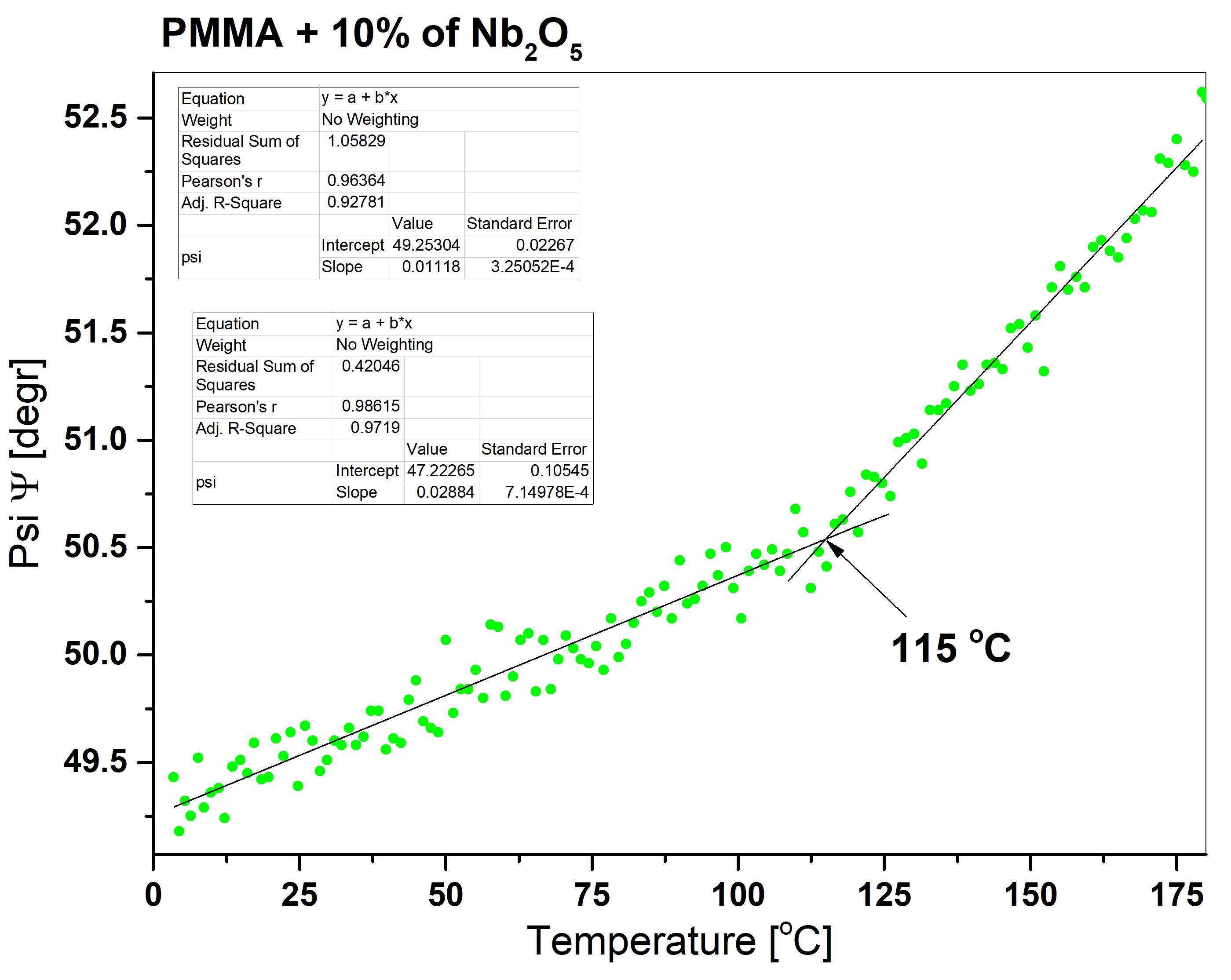


Fig. 13s Linear regressions of the ellipsometric angle Ψ as a function of temperature for the composite film PMMA/Nb_2_O_5_ (10%)


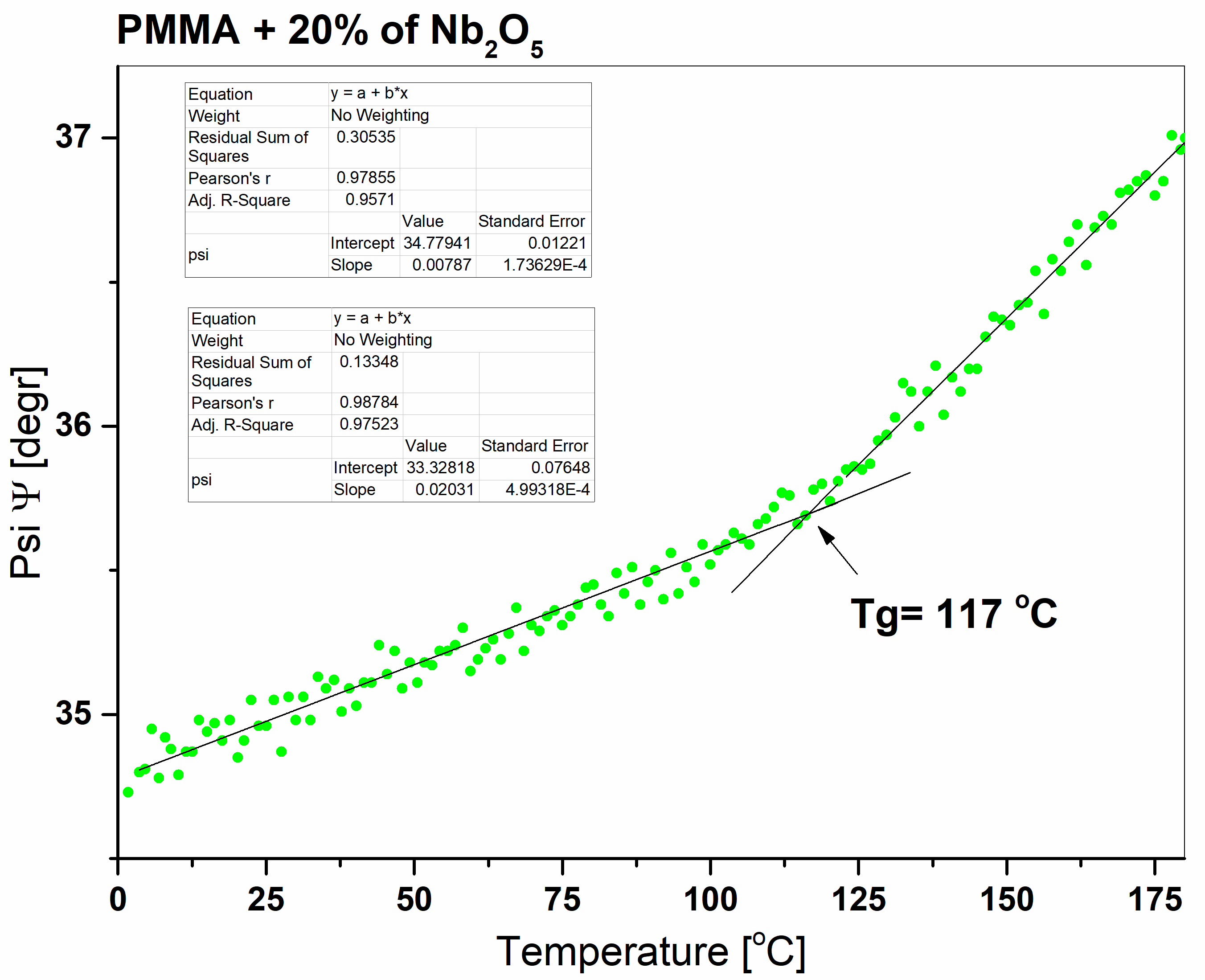


Fig. 14s Linear regressions of the ellipsometric angle Ψ as a function of temperature for the composite film PMMA/Nb_2_O_5_ (20%)

| Fit to: | Tg ± ΔTg [ᵒC] |
| --- | --- |
| pure PMMA  d(T) | 102 ± 5 |
| pure PMMA  Ψ(T) (λ=900nm) | 101 ±5 |
| PMMA/Nb_2_O_5_ (2.5%)  Ψ(T) (λ=900nm) | 115 ±5 |
| PMMA/Nb_2_O_5_ (5%)  Ψ(T) (λ=900nm) | 113 ±5 |
| PMMA/Nb_2_O_5_ (10%)  Ψ(T) (λ=900nm) | 115 ±5 |
| PMMA/Nb_2_O_5_ (20%)  Ψ(T) (λ=900nm) | 117 ±5 |

Table 1s. Glass transitions values Tg and average standard error ΔTg

**5. SEM pictures**

The pictures collected on non-annealed films are show below:


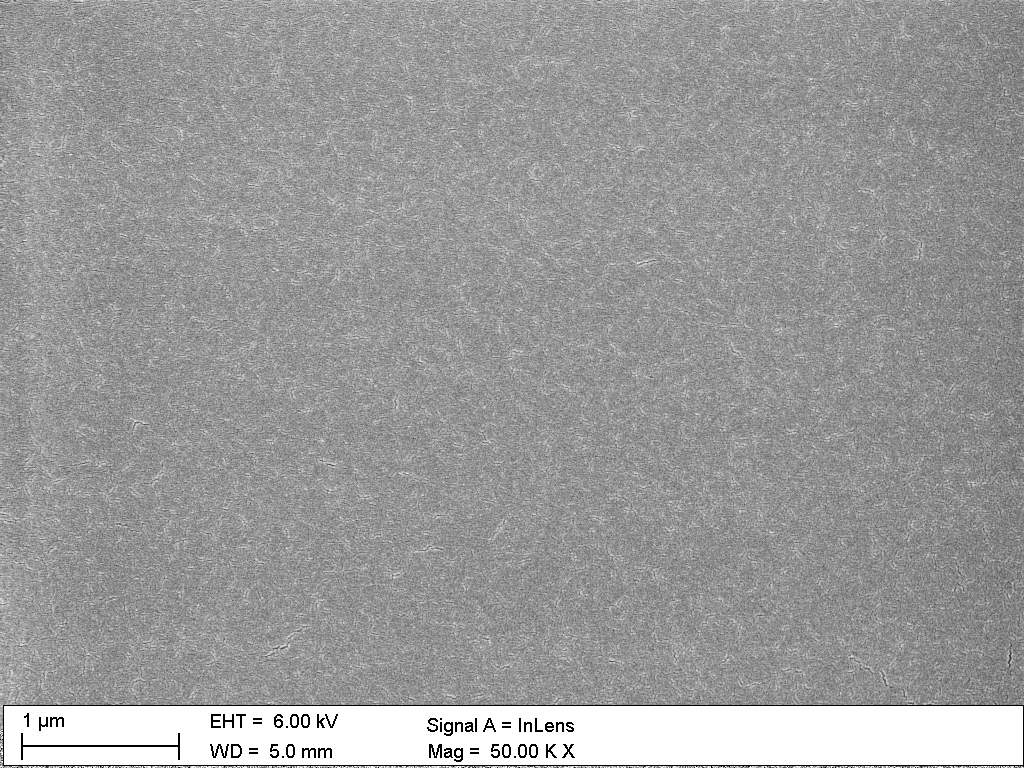


Fig. 15s Surface of pure PMMA


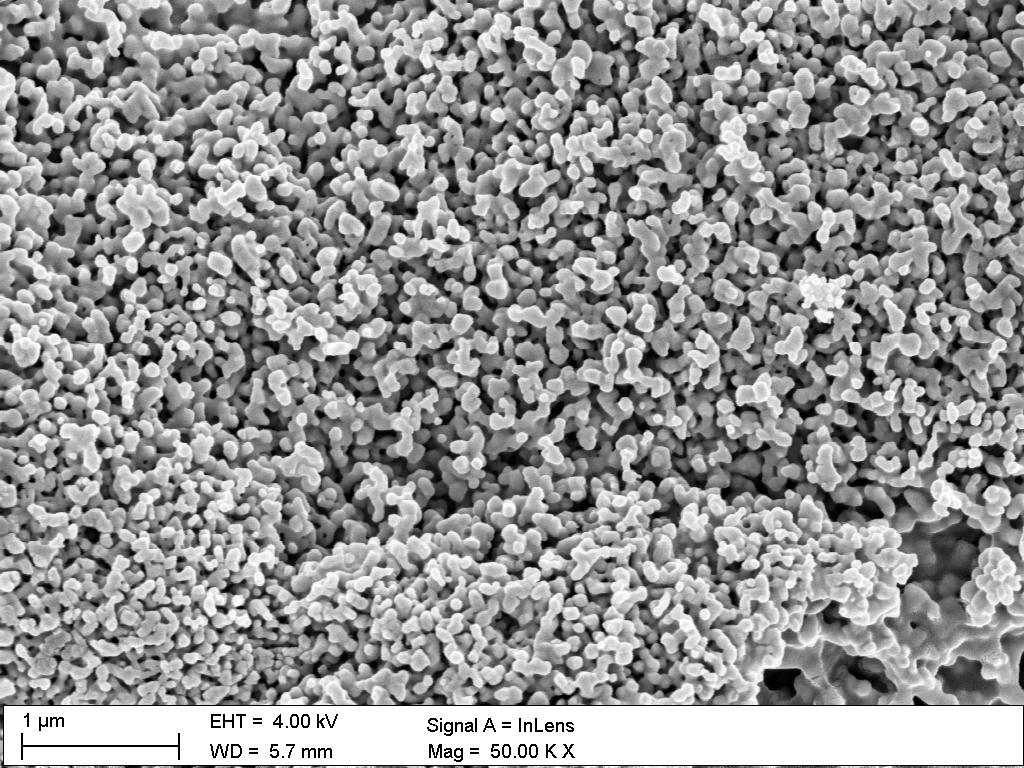


Fig. 16s Nb_2_O_5_ cluster


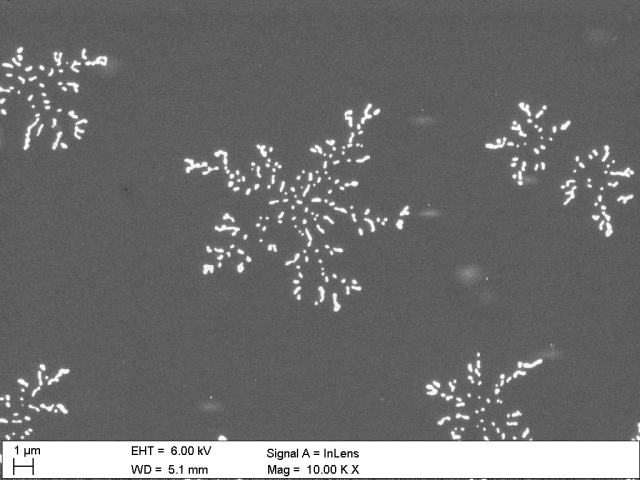


Fig. 17s Surface of PMMA/Nb_2_O_5_ films (2.5%)


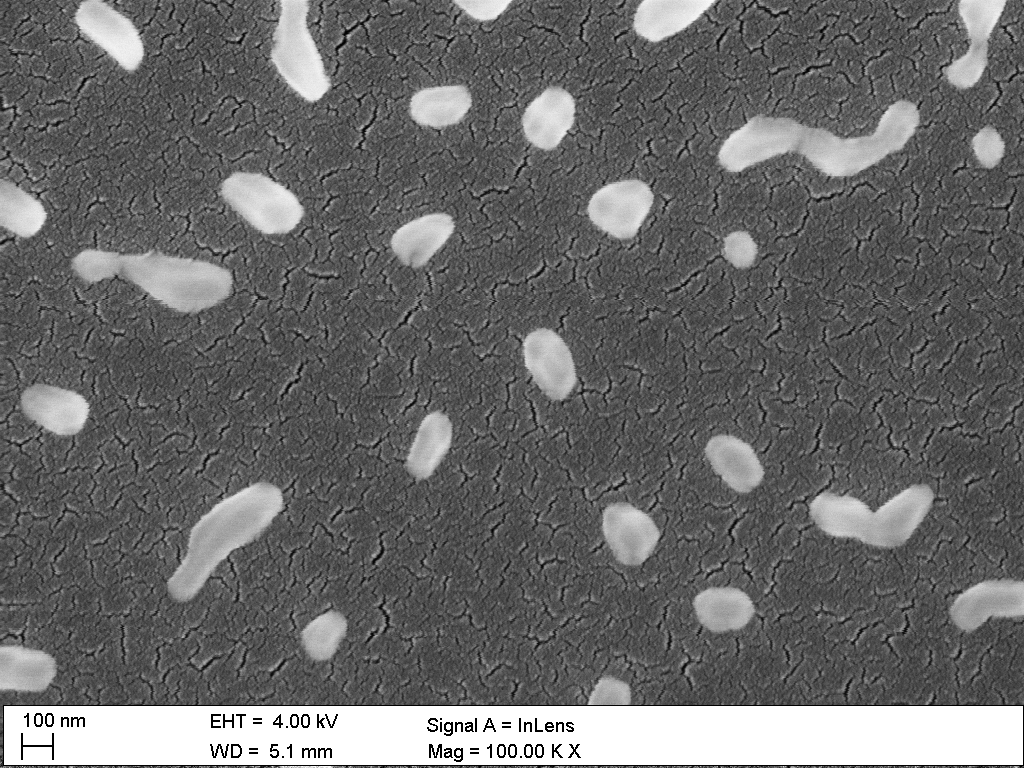


Fig. 18s Surface of PMMA/Nb_2_O_5_ film (2.5%)


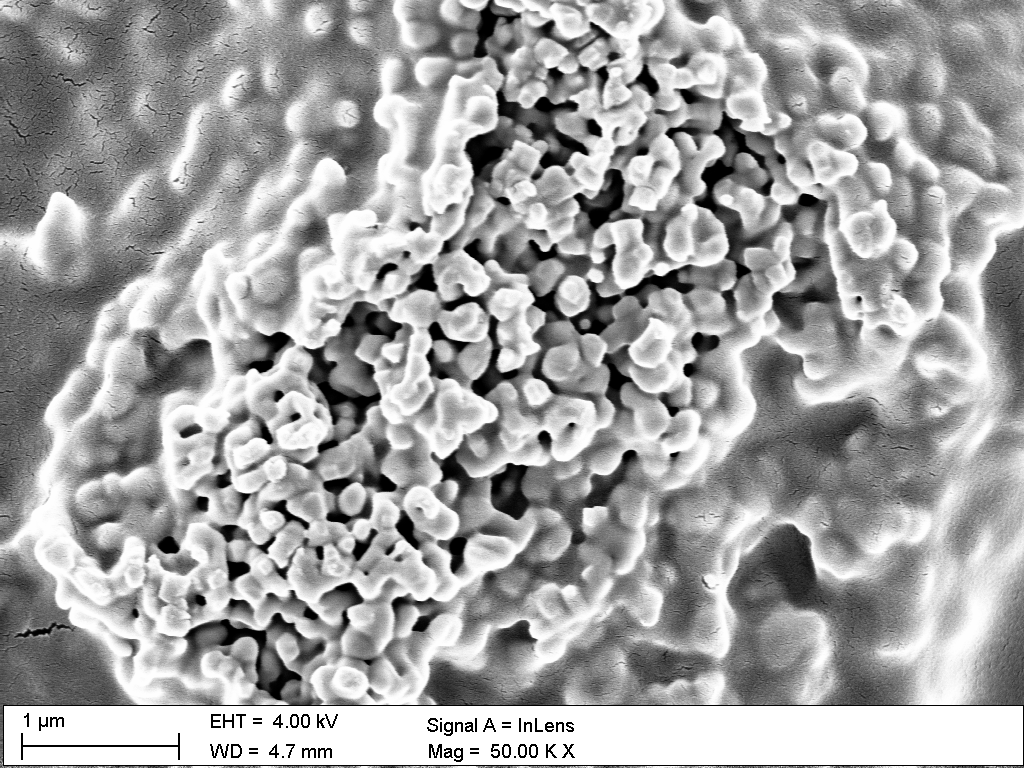


Fig. 19s Cluster on surface of PMMA/Nb_2_O_5_ film


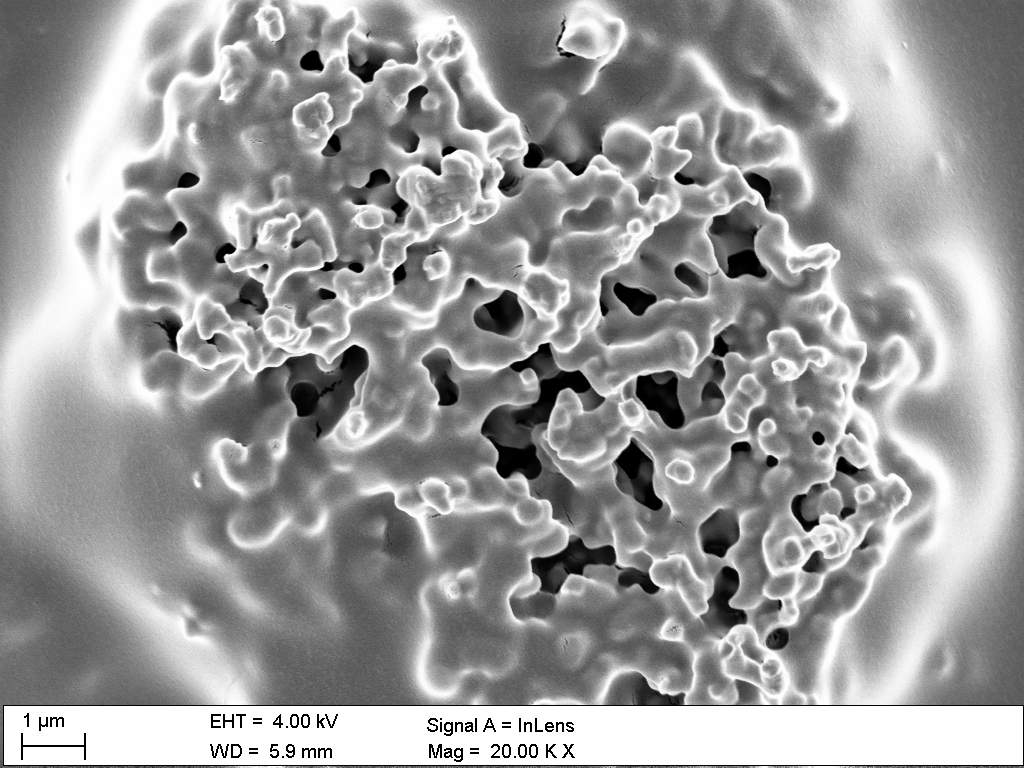


Fig. 20s Cluster on surface of PMMA/Nb_2_O_5_ film
